# Personal

**Published:** 1887-04

**Authors:** 


PERSONAL.—Our distinguished President, T. S. M. A., DR. T. H. NOTT, of Goliad, honored the JOURNAL office with a visit last week, in company with Dr. Cupples, of San Antonio. We regret to observe that the Doctor's general health is seriously impaired by want of necessary rest—incident to a large and very laborious country practice. He has gone to Waco to spend the few remaining days, ere the Convention meets : but, like the thrifty farmer, who wanted the men to "clean up the yard," while they were "resting," we fear, the Doctor will not be able to get much repose. We sincerely hope, he may be able to be with us and to give the Association one of his ablest efforts.

We are pleased to acknowledge a visit also from Dr. Garwood, of Bastrop, Drs. Heire and Burleson, of Webberville, and several other distinguished members of the profession.

